# Pan-cancer analysis reveals an immunological role and prognostic potential of PXN in human cancer

**DOI:** 10.18632/aging.203154

**Published:** 2021-06-16

**Authors:** Yun Chen, Han Zhao, Yan Xiao, Peijun Shen, Li Tan, Shaohui Zhang, Qiong Liu, Zhengrong Gao, Jie Zhao, Yaqiong Zhao, Yue Guo, Yunzhi Feng

**Affiliations:** 1 Department of Stomatology, The Second Xiangya Hospital, Central South University, Changsha, Hunan 410011, China; 2 Department of Ophthalmology, Eye, Ear, Nose, and Throat Hospital of Fudan University, Shanghai 200000, China; 3 Laboratory of Myopia, NHC Key Laboratory of Myopia, Fudan University, Chinese Academy of Medical Sciences, Shanghai 200000, China; 4 Shanghai Key Laboratory of Visual Impairment and Restoration, Fudan University, Shanghai 200000, China; 5 Nursing Department, Ganzhou Municipal Hospital, Gannan Medical University, Ganzhou, Jiangxi 341000, China; 6 Department of Gastroenterology, The Third Xiangya Hospital of Central South University, Changsha, Hunan 410011, China; 7 Hunan Key Laboratory of Nonresolving Inflammation and Cancer, Central South University, Changsha, Hunan 410011, China

**Keywords:** PXN, pan-cancer, TCGA, survival, immune infiltration

## Abstract

Paxillin (PXN) is a protein involved in numerous physiological processes, and its presence is closely related to the occurrence and development of many types of tumors. However, no studies have analyzed PXN from a pan-cancer perspective. We analyzed *PXN* expression, immune cell infiltration, prognosis, and biological function across different types of tumors included in The Cancer Genome Atlas and Gene Expression Omnibus datasets. The results showed that expression of *PXN* varies in different tumors. Expression of *PXN* strongly correlated with prognosis in patients with tumors; higher *PXN* expression usually was linked to poor overall and disease-free survival. Expression of *PXN* in breast invasive carcinoma and lymphoid neoplasm diffuse large B-cell lymphoma was related to the degree of CD8+ T-cell infiltration, and infiltration of cancer-associated fibroblasts, such as kidney renal papillary cell carcinoma and brain lower-grade glioma, was also observed in other tumors. The results of pan-cancer analysis showed that abnormal *PXN* expression was related to poor prognosis, immune infiltration, and protein phosphorylation in different tumor types. Therefore, the *PXN* gene may become a potential biomarker of clinical tumor prognosis.

## INTRODUCTION

Cancer has become one of the leading causes of death in many countries, and single-tumor-type cancer is fundamentally a genomic disease. Cancer can manifest in hundreds of different forms depending on the location of the tumor, the origin of the cells, and the spectrum of genomic changes that promote tumorigenesis and affect the response to treatment [1].

Pan-cancer analysis can reveal the correlation between any gene of interest and its clinical prognosis as well as its potential molecular mechanisms across different tumors. The Cancer Genome Atlas (TCGA) research network has aggregated and analyzed a large number of human tumors and found molecular aberrations at the DNA, RNA, protein, and epigenetic levels [2]; pan-cancer analysis could span the breadth of analyses and identify commonalities, differences, and emerging themes in human cancers [3]. With the rapid development of whole-genome sequencing technology, a large number of gene sequencing data and a big clinical database of different tumors can be obtained from online comprehensive platforms, such as TCGA, the Gene Expression Omnibus (GEO), and the Oncomine database [4, 5]. The availability of these large collections allows us to perform pan-cancer expression analysis.

Paxillin (PXN) is localized to human chromosome 12q24.31, which encodes a cytoskeletal protein that involves actin-membrane attachment to the extracellular matrix [6, 7]. Protein-protein interactions and phosphorylation analysis have demonstrated that the PXN protein is involved in a variety of physiological processes, such as matrix organization, focal adhesion assembly and disassembly, tissue remolding, cell proliferation and survival, cell motility, and metastasis [8, 9]. Previous studies have shown functional links between PXN and tumorigenesis, such as oral cavity squamous cell carcinoma [10], gastric cancer [11], lung carcinoma [6], colorectal cancer [12], glioblastoma [13], breast cancer [14], and renal cell carcinoma [15]. The high expression of PXN in certain tumors is related to tumor stage, poor differentiation, lymphatic vascular invasion, lymphatic metastasis, distant metastasis, tumor lymph node metastasis staging, and recurrence in distant sites after radical surgery [16, 17]. However, the human pan-cancer evidence of a potential role for the *PXN* gene in various tumor types remains unclear.

Given the crucial role of PXN in tumorigenesis, we conducted a pan-cancer analysis of *PXN* expression and patient prognoses via TCGA, the Clinical Proteomic Tumor Analysis Consortium (CPTAC), the Human Protein Atlas (HPA) cohort, and GEO databases. In addition, we explored the correlations between *PXN* expression and protein phosphorylation, immune infiltration, and genetic mutation. Our findings indicated statistical correlations of *PXN* gene expression with clinical prognosis, protein phosphorylation, immune infiltration, and genetic mutation, which suggests that PXN is a potential prognostic biomarker.

## RESULTS

### Gene expression analysis of *PXN*

**Figure 1:**
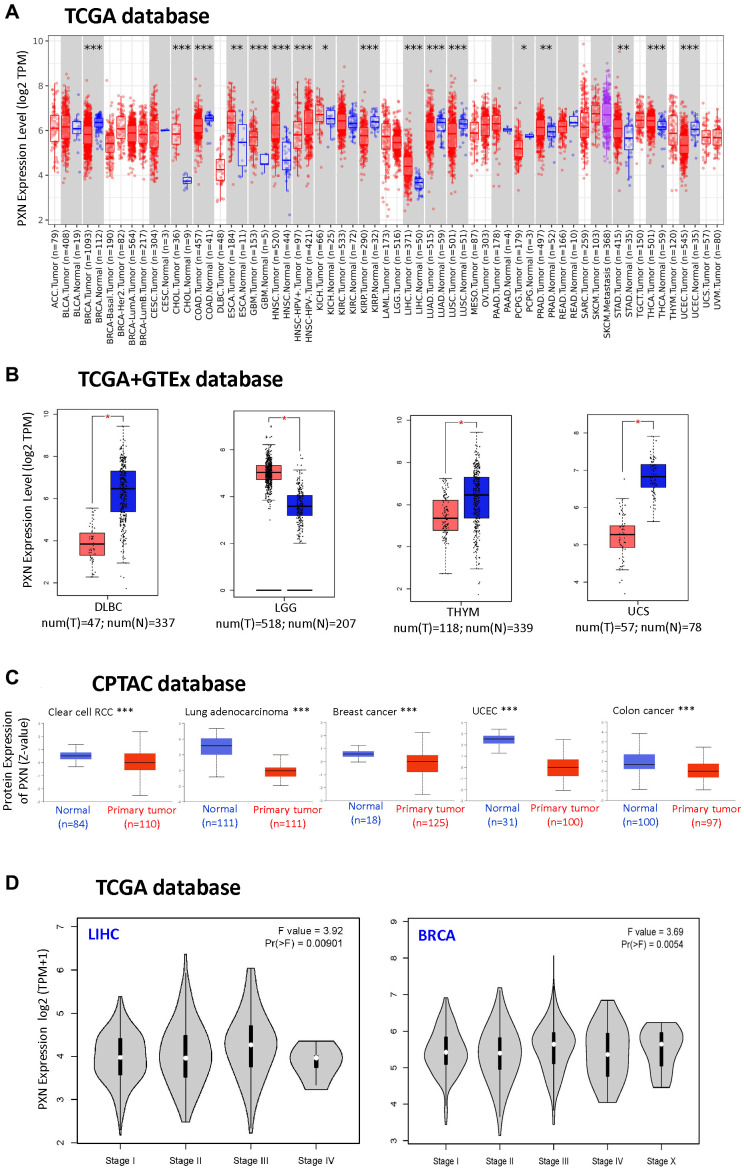
*PXN* gene expression in different tumors and pathological stages (**A**) using TIMER2 to analyze the expression of *PXN* in different cancers or specific cancer subtypes (^*^P < 0.05; ^**^P < 0.01; ^***^P < 0.001); and (**B**) using the box plot data to analyze the type of lymphoid neoplasm DLBC, LGG, thymoma, and uterine carcinosarcoma in TCGA, for which the corresponding normal tissues of the GTEx database were included as controls (^**^P < 0.01). (**C**) Using the CPTAC dataset, PXN total protein expression levels in normal tissue versus primary tissue were analyzed for RCC, lung adenocarcinoma, breast cancer, UCEC, and colon cancer (^***^P < 0.001). (**D**) Using TCGA data, *PXN* gene expression was analyzed by main pathological stage (stage I, stage II, stage III, and stage IV) of LIHC and BRCA. Log_2_ (TPM + 1) was used for the log scale.

To compare expression levels of the *PXN* gene between tumor and normal tissues, we obtained expression of *PXN* across various cancer types in TCGA dataset via the TIMER2 tool. As shown in Figure 1A, *PXN* expression in human tumors of cholangiocarcinoma, esophageal carcinoma, glioblastoma multiforme (GBM), head and neck squamous cell carcinoma (HNSC), liver hepatocellular carcinoma (LIHC), thyroid carcinoma (*P* < 0.001), prostate adenocarcinoma (PRAD), stomach adenocarcinoma (*P* < 0.01), and kidney chromophobe (*P* < 0.05) were higher than expressions in paired normal tissues. However, *PXN* expression in breast invasive carcinoma (BRCA), colon adenocarcinoma (COAD), kidney renal papillary cell carcinoma, lung adenocarcinoma (LUAD), lung squamous cell carcinoma (LUSC), uterine corpus endometrial carcinoma (UCEC) (*P* < 0.001), and pheochromocytoma and paraganglioma (*P* < 0.05) were significantly downregulated. The differential expression of *PXN* in different tumor types suggested that PXN has various regulatory mechanisms in different tumor types.

To support a correlation of *PXN* expression levels between tumor and normal tissues, we used GEPIA2, which analyzed our target genes from TCGA and in the GTEx projects. *PXN* expression was downregulated in lymphoid neoplasm diffuse large B-cell lymphoma (DLBC), thymoma, and uterine carcinosarcoma (*P* < 0.01). However, *PXN* expression was upregulated in brain lower-grade glioma (LGG) (*P* < 0.01, Figure 1B).

Next, we used the CPTAC dataset to study the protein level of PXN between different tumor types and normal tissues. We found that the PXN mRNA level was downregulated in renal clear cell carcinoma (RCC), LUAD, BRCA, COAD, and UCEC (*P* < 0.001) compared with levels in normal tissue controls (Figure 1C).

We also observed a correlation between *PXN* expression level and the pathological stages of cancers, including in BRCA and LIHC (*P* < 0.01) via the “Pathological Stage Plot” module of GEPIA2 (Figure 1D).

### The prognostic value of PXN

**Figure 2:**
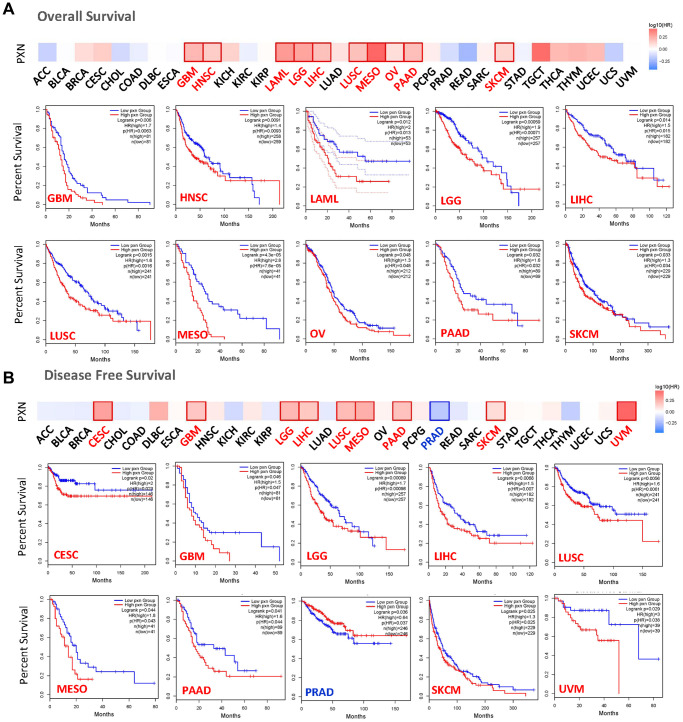
**The relationship between *PXN* gene expression and survival prognosis of cancers in TCGA.** The (**A**) overall survival rate and (**B**) disease-free survival rate, as well as *PXN* gene expression in different tumors in TCGA, were analyzed by GEPIA2 software. The survival diagram and Kaplan-Meier curves with positive results are shown.

We used the dataset from TCGA and GEPIA to investigate the correlation of *PXN* expression with prognoses of patients across different tumor types. Upregulated *PXN* expression was linked to poor prognosis and OS in many cancers, including GBM, HNSC, acute myeloid leukemia, LGG, LIHC, LUSC, mesothelioma (MESO), ovarian cancer, pancreatic adenocarcinoma (PAAD), and skin cutaneous melanoma (SKCM) (Figure 2A). In addition, upregulated *PXN* expression was linked to poor DFS in different cancer types, including CESC, GBM, LGG, LIHC, LUSC, MESO, PAAD, SKCM, and uveal melanoma (UVM). Downregulated *PXN* expression was linked to poor DFS in PRAD (Figure 2B).

**Figure 3:**
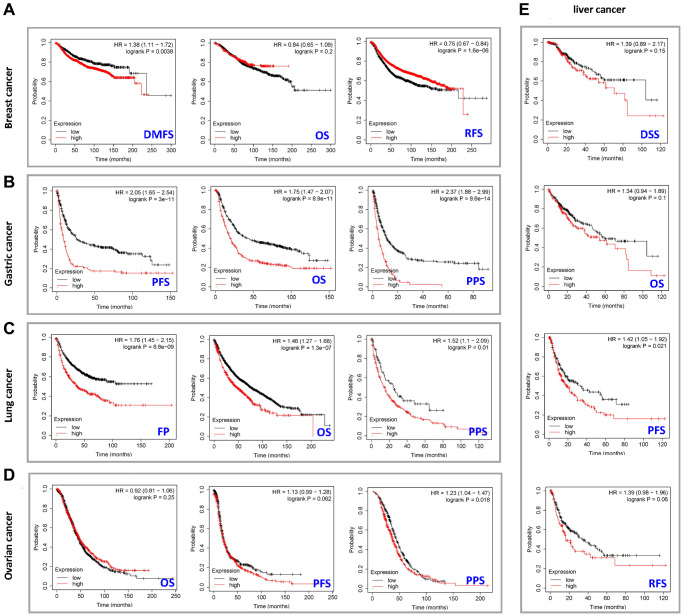
**Correlation between *PXN* gene expression and prognosis of cancers using Kaplan-Meier curves.** We used the Kaplan-Meier plotter to perform a series of survival analyses, including DMFS, OS, RFS, DSS, PFS, post-PPS, and first progression, reflecting *PXN* gene expression in (**A**) breast cancer, (**B**) gastric cancer, (**C**) lung cancer, (**D**) ovarian cancer, and (**E**) liver cancer.

Next, we studied the correlation between expression level of the *PXN* gene and cancer prognoses by using the Kaplan-Meier plotter. Notably, high *PXN* gene expression was significantly correlated with poor DMFS in breast cancer (*P* < 0.01). However, a low *PXN* expression level correlated with poor RFS in breast cancer (*P* < 0.001) (Figure 3A). Highly expressed *PXN* was linked to poor PFS, OS, and PPS in gastric cancer (*P* < 0.001) and poor PS (*P* < 0.001), OS (*P* < 0.001), and PPS (*P* = 0.01) in lung cancer (Figure 3B and 3C). Moreover, highly expressed *PXN* was linked to poor PPS in ovarian cancer (*P* < 0.05) and poor PFS (*P* < 0.001) in liver cancer (Figure 3D and 3E).

### *PXN* mutation in various tumors

**Figure 4:**
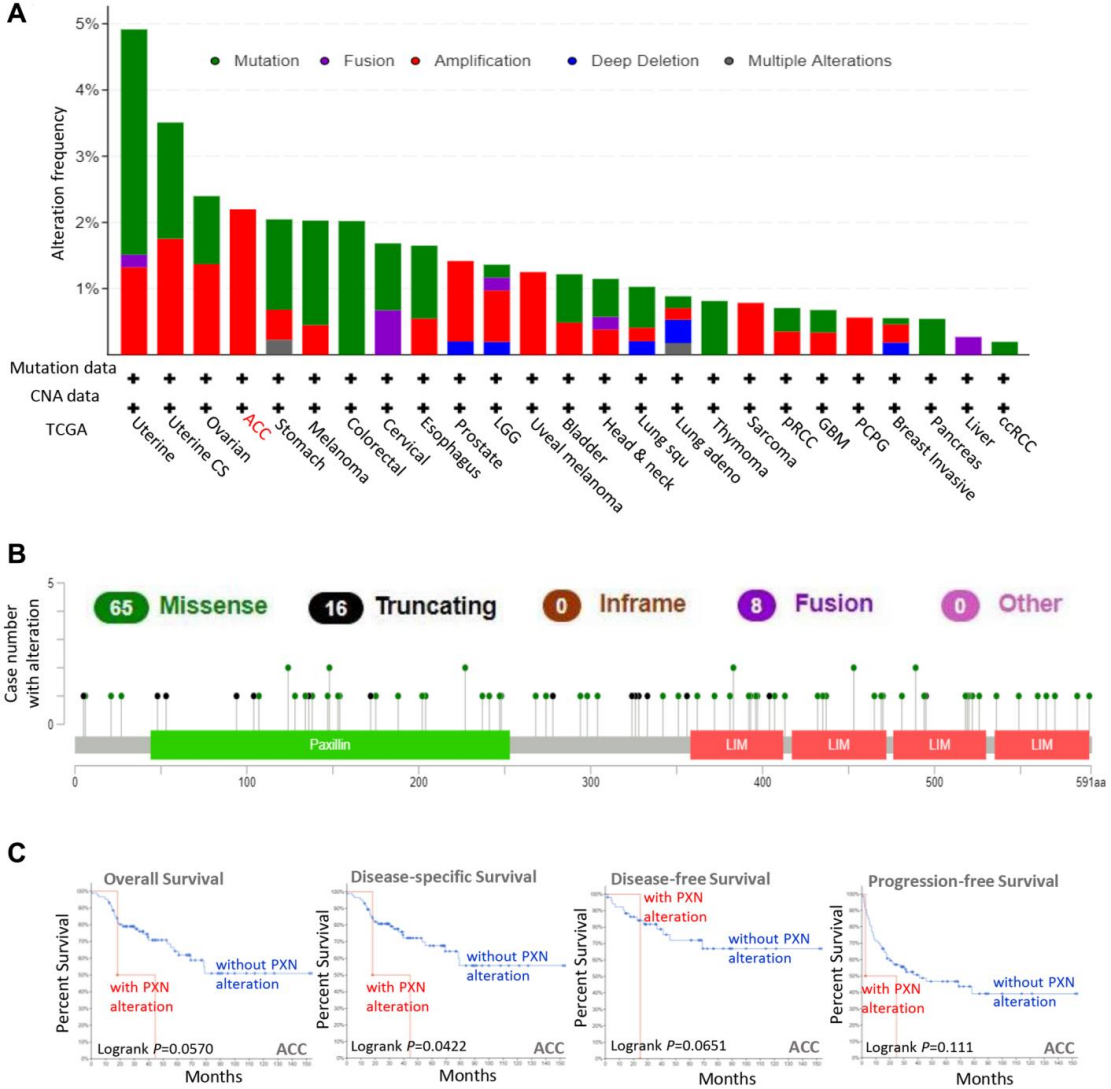
**Mutation feature of *PXN* in different cancers in TCGA.** Using the cBioPortal tool, we analyzed the mutation features of *PXN* for tumors in TCGA. The alteration frequencies with (**A**) mutation type and (**B**) mutation site are shown. (**C**) Using the cBioPortal tool, we analyzed the potential correlation between mutation status and overall, disease-specific, disease-free, and progression-free survivals of ACC.

To study the relevance of the *PXN* gene mutation across various human cancers, we used the cBioPortal tool to detect *PXN* mutations in data extracted from TCGA dataset. As shown in Figure 4A, *PXN* has the highest mutation frequency in patients with uterine tumors (nearly 5%). The mutation frequency of the *PXN* gene in patients with uterine carcinosarcoma is nearly as high. Notably, all patients with adrenocortical carcinoma (ACC) had amplification of the *PXN* gene, which showed an alteration frequency of ~2%. All data about types, sites, and case numbers of the *PXN* genetic alteration—including data about missense, truncating, and fusion mutations—are presented in Figure 4B. Missense mutation of *PXN* was the main type of genetic alteration. Moreover, the clinical survival prognosis value of *PXN* alterations reflected better prognosis in patients with ACC with regard to DSS (*P* = 0.0422), but not OS (*P* = 0.057), DFS (*P* = 0.0651), or PFS (*P* = 0.111) (Figure 4C).

### Protein expression analysis of PXN

**Figure 5:**
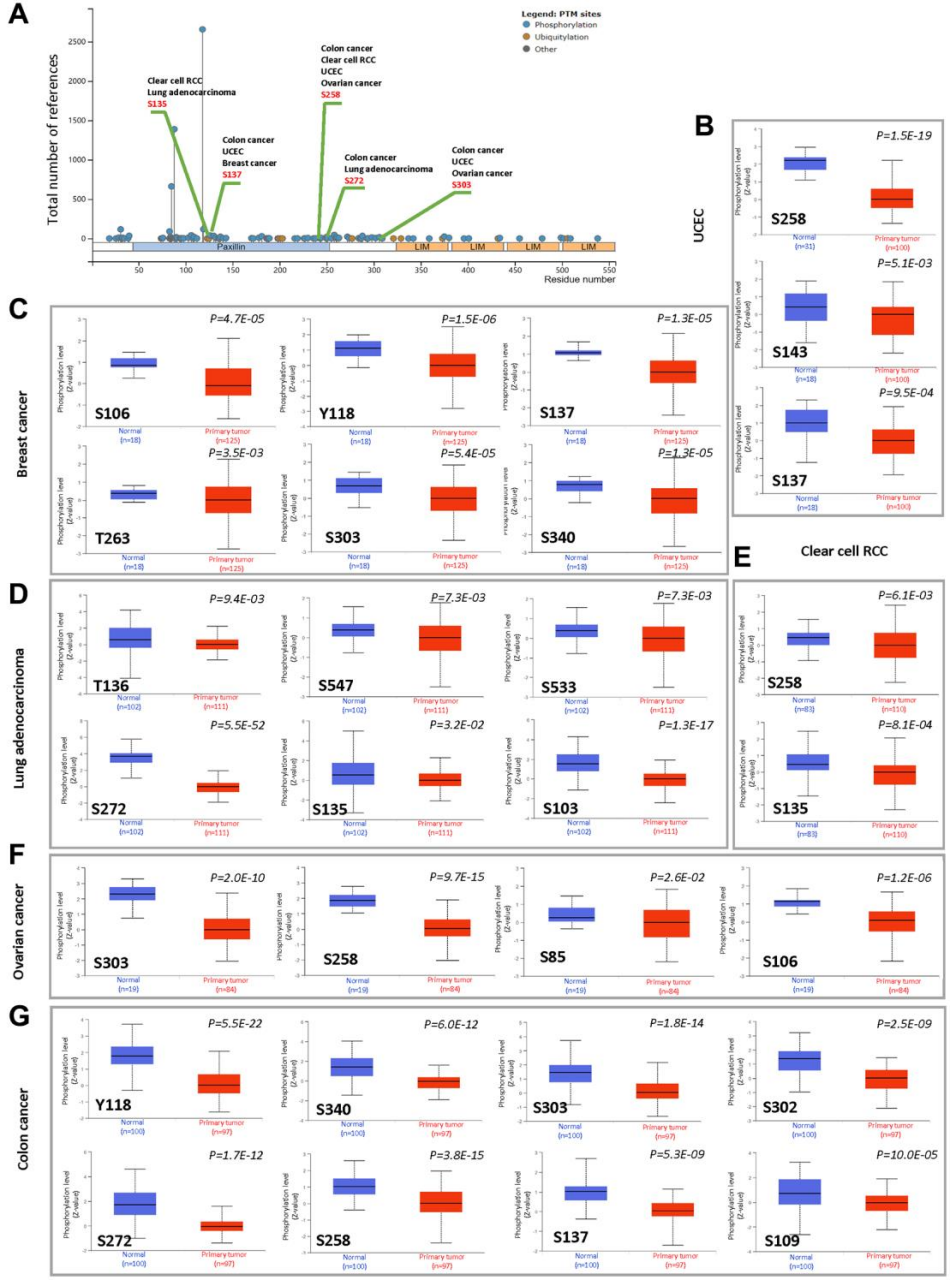
**Phosphorylation analysis of the PXN protein in different cancers.** Using the CPTAC dataset, we analyzed the expression level of PXN phosphoproteins (S135, S137, S258, S272, S303, S106, Y118, T263, S340, S143, S136, S547, S533, S272, S103, S85, S302, and S109 sites) between normal tissue and primary tissue of selected tumors with UALCAN. (**A**) Schematic diagram of PXN protein showing phosphoprotein sites with positive results. Box plots are shown for different cancers: (**B**) UCEC, (**C**) breast cancer, (**D**) lung adenocarcinoma, (**E**) RCC, (**F**) ovarian cancer, and (**G**) colon cancer.

We used the CPTAC dataset to investigate the differences in protein phosphorylation of PXN between tumor tissues and normal tissues across six types of tumors (BRCA, RCC, LUAD, ovarian cancer, colon cancer and UCEC). Figure 5A presents the sites of protein phosphorylation on PXN. After a series of analyses, we observed that four types of tumor (colon cancer, RCC, UCEC, and ovarian cancer) exhibit a lower phosphorylation level in all primary tumor tissues compared with normal tissues at the S258 locus within the PXN domain (*P* < 0.001). Lower protein phosphorylation of PXN was noted in colon cancer, UCEC, and breast cancer at the S137 locus within the PXN domain (*P* < 0.001), followed by a decreased phosphorylation level of the S303 locus for colon cancer, UCEC, and ovarian cancer (*P* < 0.001) (Figure 5B–5G).

**Figure 6:**
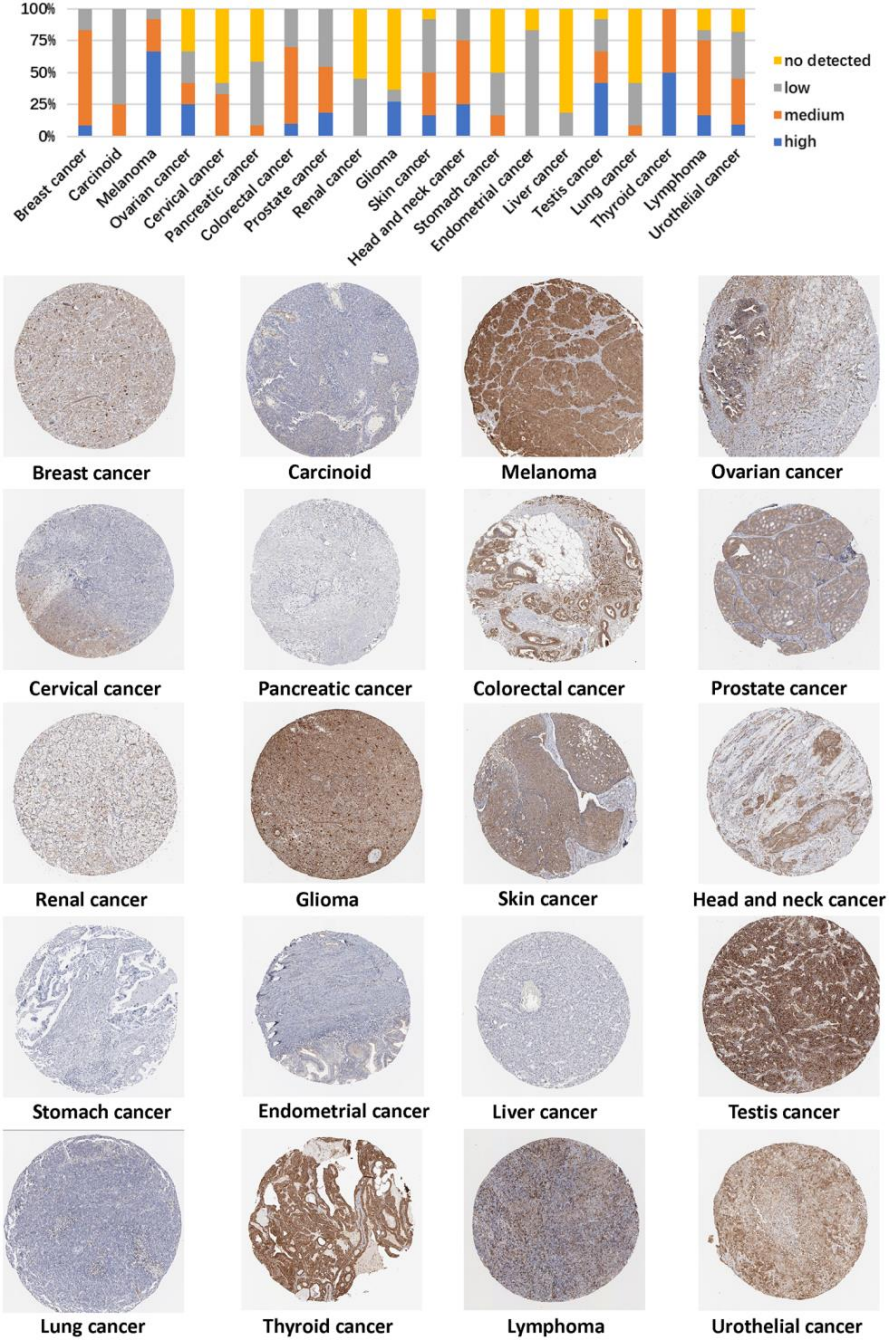
Representative immunohistochemical staining results of the PXN protein in different cancer tissues.

We also used the HPA cohort to investigate PXN protein expression levels in various types of cancer. Analysis showed that aberrant expression of *PXN* was detected in 20 types of tumor tissues. High *PXN* expression levels were observed in UVM (66.7%), thyroid carcinoma (54%), testicular germ cell tumors (41.7%), GBM (27.3%), HNSC (25%), ovarian cancer (25%), PRAD (18.2%), DLBC (16.7%), SKCM (16.7%), COAD (10%), BLCA (9.1%), and BRCA (9.1%) (Figure 6). These findings could indicate in part that PXN plays different roles in various cancers.

### Immune cell infiltration of PXN in patients with cancer

**Figure 7:**
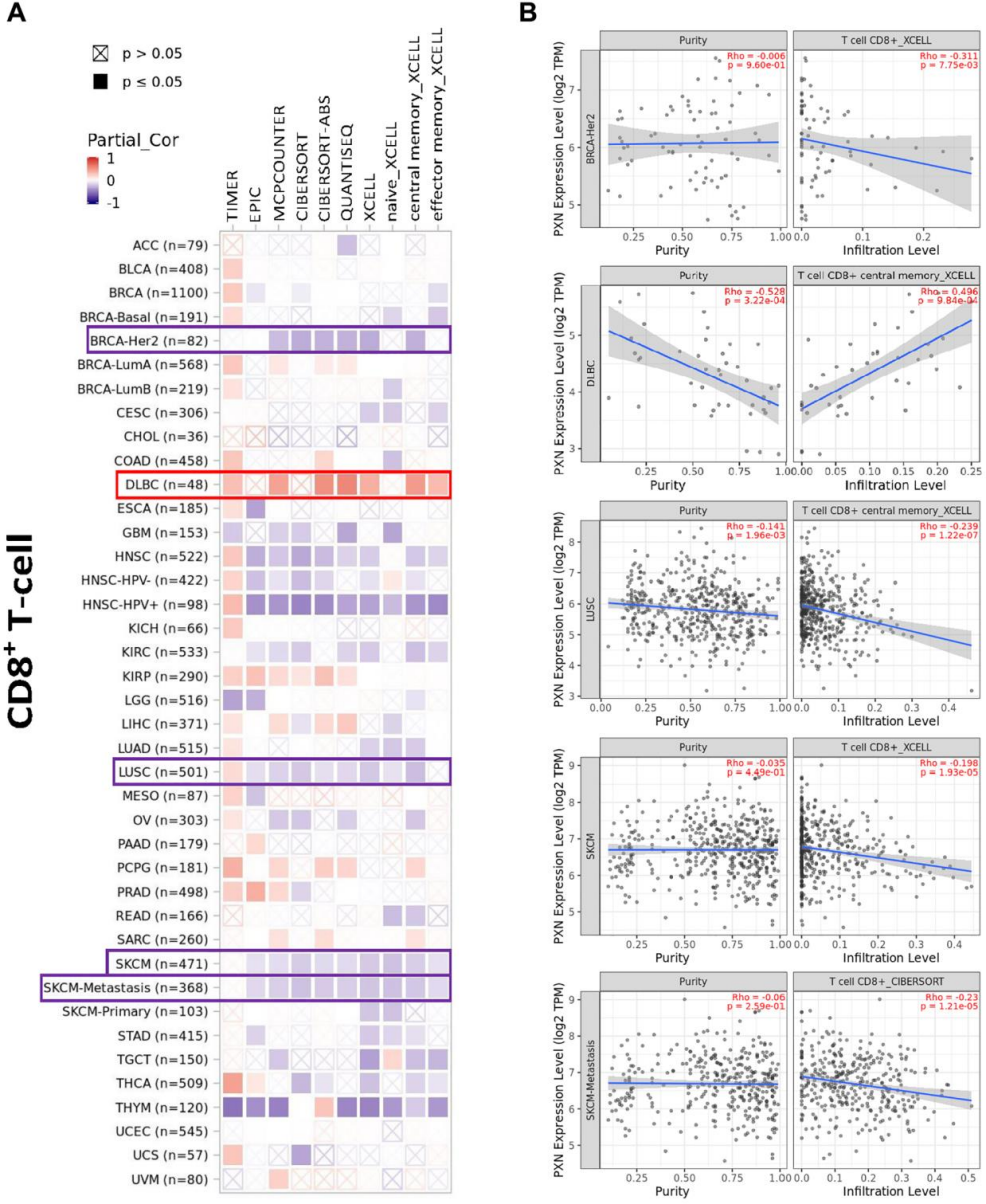
**Correlation analysis between *PXN* gene expression and immune infiltration of CD8+ T cells.** Different algorithms explored potential correlations in (**A**) *PXN* expression level and (**B**) infiltration of CD8+ T cells across all types of cancer in TCGA.

PXN is involved in immune cell infiltration and inflammatory responses, which play key roles in initiation, progression, and metastasis of tumors. Therefore, we used TIMER2, EPIC, MCPCOUNTER, CIBERSORT, CIBERSORT-ABS, QUANTISEQ, XCELL, naïve_XCELL, central memory_XCELL, and effector memory_XCELL algorithms to perform a comprehensive exploration of the correlation between immune cell infiltration and differential expression of *PXN* across diverse cancer types from TCGA. A negative correlation was observed between *PXN* expression and infiltration of CD8+ and human epidermal growth factor receptor 2-negative T cells in BRCA, LUSC, SKCM, and SKCM metastasis. A positive correlation was observed between *PXN* expression and infiltration of CD8+ T cells in DLBC (Figure 7).

**Figure 8:**
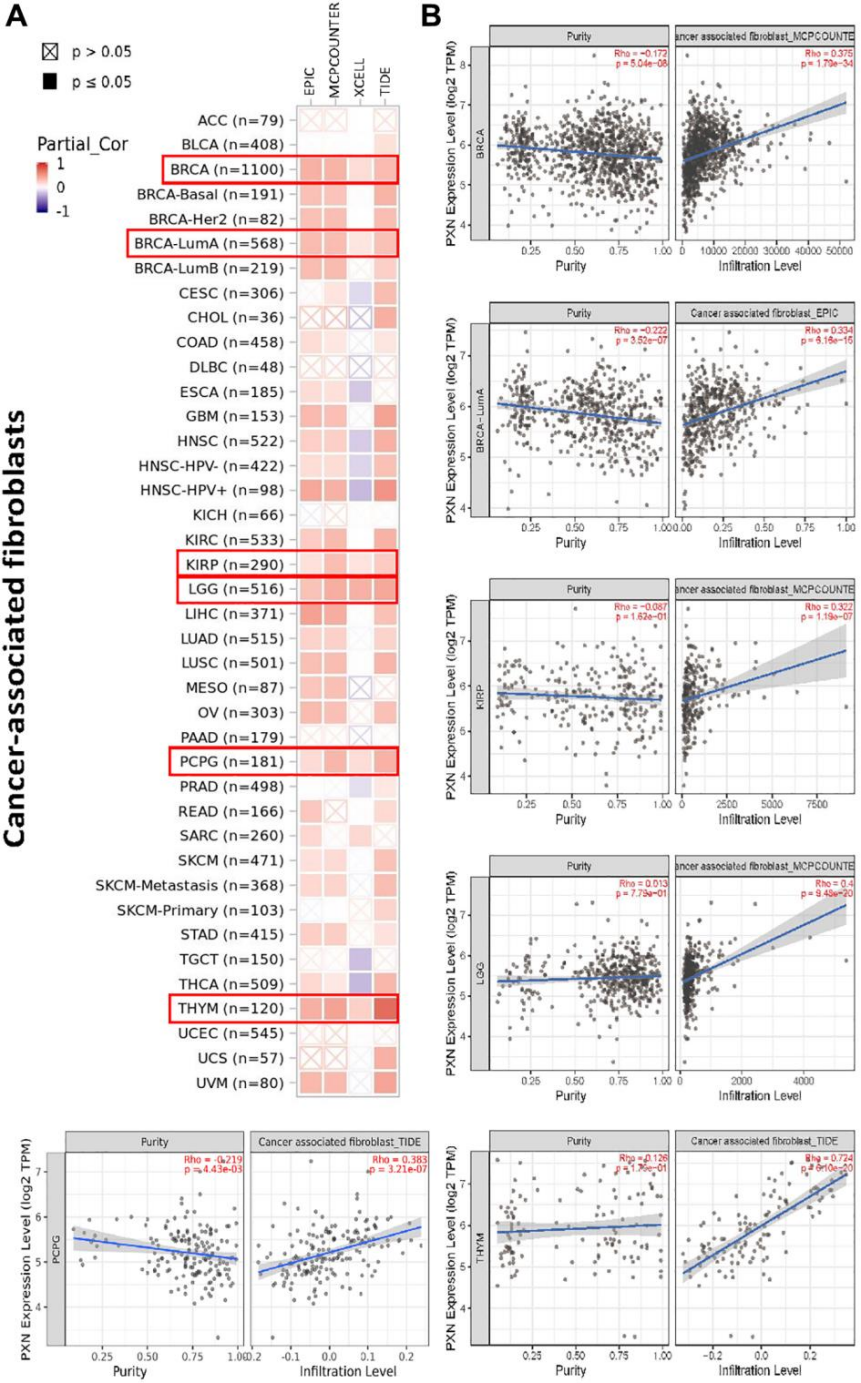
**Correlation analysis between *PXN* gene expression and immune infiltration of cancer-associated fibroblasts.** Different algorithms explored the potential correlations of (**A**) expression of the *PXN* gene and (**B**) infiltration of cancer-associated fibroblasts across all types of cancer in TCGA.

Moreover, we also detected a correlation between *PXN* expression and tumor-infiltrating immune cells in cancer-associated fibroblasts. *PXN* expression positively correlated with the infiltration level in BRCA, kidney renal papillary cell carcinoma, LGG, pheochromocytoma and paraganglioma, and thymoma (Figure 8).

### Functional enrichment analysis of PXN

**Figure 9:**
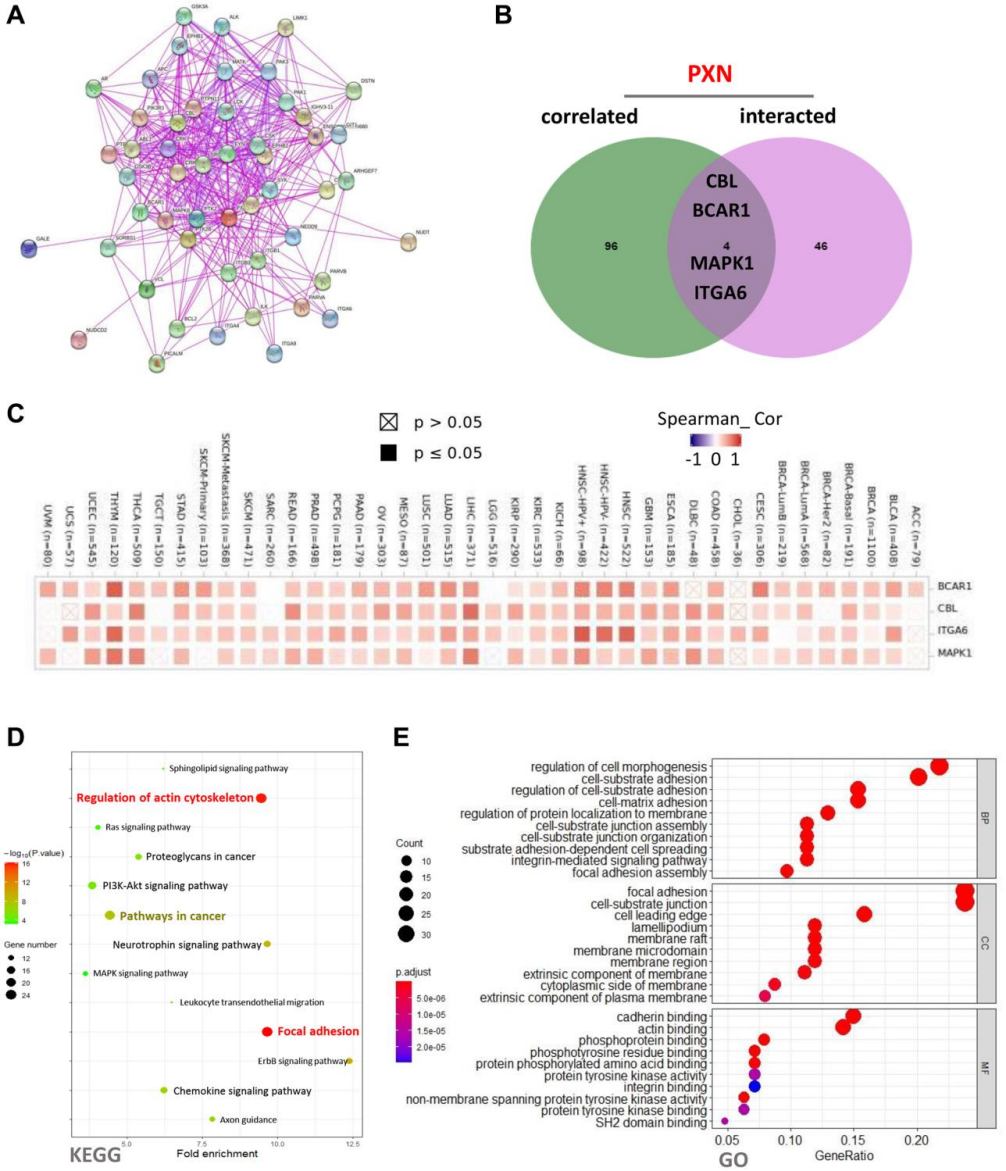
*PXN*-related gene enrichment analysis (**A**) using the STRING tool to obtain the available experimentally determined PXN-binding proteins; (**B**) as an intersection analysis of *PXN*-binding and correlated genes; and (**C**) as a corresponding heatmap of the detailed cancer types. (**D**–**E**) KEGG pathway analysis of *PXN*-binding and interacting genes, represented by a dot plot of the biological process, the cellular component, and the molecular function data in the GO analysis.

Using the STRING online database, we attempted to obtain a network of 50 PXN-binding protein interactions. The network was based on experimental evidence and had 51 nodes and 395 edges. As shown in Figure 9A, the nodes represent genes, and the edges represent the links between binding genes. We then used the GEPIA2 tool to identify the top 100 genes that correlated with *PXN* expression. Then, we used a Venn diagram to analyze the interaction of the two groups that showed four common members: *CBL*, *BCAR1*, *MAPK1*, and *ITGA6* (Figure 9B). According to the heatmap data, there was a positive correlation between *PXN* expression and the four selected genes (Figure 9C).

To further investigate the functional and pathway enrichment analyses of PXN, we used the DAVID 6.8 online tool to perform KEGG and GO enrichment analyses. As shown in Figure 9D, KEGG pathways analysis indicated that regulation of the actin cytoskeleton and focal adhesions might play important roles in connecting PXN with tumor initiation and progression. In addition, GO enrichment analysis data suggested that most of these genes are linked to regulation of cell morphogenesis in the BP category, focal adhesion in the CC category, and cadherin binding in the MF category (Figure 9E and Supplementary Figure 1).

## DISCUSSION

It has been reported that *PXN* is a highly conserved gene, which has been identified in 168 organisms [18]. The human *PXN* gene has four isoforms, and the outcome of alternative splicing, and the gene exerts important roles in focal adhesion, tumor progression and migration, barrier dysfunction of endothelial cells, inflammation, and oxidative stress [19]. PXN, as an important part of the focal adhesion complex, is correlated with poor clinical outcomes in patients with tumors [20, 21]. Meanwhile, many studies have reported that aberrant *PXN* expression is often found in relation to the initiation, progression, or metastasis of human cancer [22]. However, the roles of PXN in human pan-cancer are not well understood. In this study, we identified the relationship of the *PXN* gene in multiple cancer tumorigenesis models via a pan-cancer analysis of TCGA, CPTAC, HPA cohort, and GEO databases.

According to the Oncomine dataset, TCGA dataset, and Kaplan-Meier plotter, the gene expression analysis suggests that abnormal expression of *PXN* occurred frequently in various types of cancer. We found that *PXN* expression was upregulated in human tumors (cholangiocarcinoma, esophageal carcinoma, GBM, HNSC, LIHC, thyroid carcinoma, PRAD, stomach adenocarcinoma, and kidney chromophobe) compared with corresponding normal tissues. However, PXN expression was downregulated in BRCA, COAD, kidney renal papillary cell carcinoma, LUAD, LUSC, UCEC, and pheochromocytoma and paraganglioma. Therefore, discrepancies of *PXN* expression in various cancers indicated that PXN may have different biological functions in different types of cancer. Regardless, aberrant expression levels of PXN were associated with poor prognoses in many types of cancer, which strongly indicates that *PXN* is a potential prognostic biomarker in patients with cancer.

The tumor microenvironment (TME) is a tumor-promoting setting that tumor cells use to evade immune surveillance. The presence of the TME significantly influences therapeutic response and clinical outcome. Previous studies have identified various components that participate in the formation of the TME, including cancer-associated fibroblasts, lymphocytes, endothelial cells, mesenchymal stem cells, and the extracellular matrix [23–26]. PXN, as a main component of focal adhesions, plays an important role in the extracellular matrix [19]. PXN may also have an important effect on the TME and on immune response. In this study, we investigated the correlation between the *PXN* gene and immune cells as well as stromal infiltration with the CIBERSORT, CIBERSORT-ABS, QUANTISEQ, XCELL, MCPCOUNTER, and EPIC algorithms. *PXN* expression had functions associated with CD8+ T-cell infiltration, cancer-associated fibroblasts, and endothelial cells (Supplementary Figure 2) in different tumors. CD8+ T cells play an important role in immune-related tolerance and immunosuppression within the TME [27]. Cancer-associated fibroblasts and endothelial cells serve pro-tumorigenic roles in the TME via secretion of various growth factors, cytokines, and chemokines and via degradation of the extracellular matrix [28, 29]. This association between PXN and TME might be another reason for the prognostic implications of PXN in various cancers. We failed to detect a correlation between *PXN* expression and mesenchymal stem cells, monocytes, or myeloid-derived suppressor cells. Several studies have reported that PXN plays an important role in macrophage migration and phagocytosis [30]. Taken together, these results suggest that aberrant *PXN* expression might play a leading role in the TME.

Gene mutations play an important role in the pathogenesis of some cancers [31]. In this study, the most frequent DNA alterations of the *PXN* gene in the dataset from TCGA were missense mutations. Specific gene mutations may predict patient prognosis and treatment response. We used the CPTAC dataset to investigate the function and localization of the PXN protein in various cancers. We observed that PXN is phosphorylated at multiple residues, which are important for PXN protein interactions with downstream signaling and adapter proteins. Recent data show that degradation or turnover of local adhesion complexes is also regulated by serine phosphorylation during migration [32]. In addition, tyrosine phosphorylation of the PXN protein might involve regulation of both the degradation and turnover of local adhesion complexes [33]. The sites of protein phosphorylation on PXN might be potential targets for cancer treatment. However, the precise biological mechanism underlying serine or tyrosine phosphorylation of PXN to drive the regulation of adhesion dynamics and migration is not yet fully investigated.

We sought to characterize the function of differentially expressed PXN via GO enrichment analysis and KEGG pathway enrichment analysis. We found that differentially expressed PXN was mainly associated with regulation of the actin cytoskeleton, focal adhesions, pathways in cancer, and the PI3K-AKT signaling pathway. Importantly, the focal adhesion kinase/PXN pathway plays a crucial role in cancer cell migration by regulating small Rho GTPases [34]. Previous work in colon cancer has shown that phosphatase and tensin homolog can inhibit *PXN* expression via PI3K/AKT/NF-kB signaling [35]. Previous studies have demonstrated that PXN plays key roles in multiple receptor-activated signaling pathways in breast cancer metastasis, taking part in cell transformation and migration [36]. According to the protein-protein interaction analysis, *CBL*, *BCAR1*, *MAPK1*, and *ITGA6* were predicted to positively correlate with the *PXN* gene. This provides a hint that PXN-related enrichment pathways could serve as underlying markers for patients to help determine therapy.

Previous data confirmed that *PXN* expression was positively associated with the epithelial-mesenchymal transition process in different types of tumors, including colorectal cancer, RCC, and triple-negative breast cancer. PXN knockdown significantly inhibited migration and invasion of cancer cell lines by interfering with the epithelial-mesenchymal transition process [14–16]. We also observed a positive correlation between *PXN* expression and survival prognoses in colorectal cancer, RCC, and BRCA. Thus, epithelial-mesenchymal transition might be another reason for the different prognostic implications of PXN in colorectal cancer, RCC, and BRCA.

In this study, we investigated the pan-cancer analysis of PXN in various cancers and explored the association of its aberrant expression with patient survival outcomes. This study has several limitations. First, although TCGA, GTEx, and CPTAC datasets were included in this study, the numbers of each cancer type were still limited. Data about some particular cancer types were not available. Second, given the myriad individual differences among patients with cancer, it was difficult to cover all possible variations in this study. Finally, this study was only based on bioinformatics and relied on public databases. Future mechanistic studies to validate the expression and function of PXN at the cellular and molecular levels are needed.

In conclusion, the results of this pan-cancer analysis indicated that aberrant expression of *PXN* correlates with poor prognosis, immune cell infiltration, and protein phosphorylation in different cancer types. In addition, these data provided the landscape of comprehensive features of PXN in pan-cancer tumor types. The results of this study suggest that the *PXN* gene is a potential prognostic biomarker for clinical diagnosis and assessment of tumors.

## MATERIALS AND METHODS

### TIMER2

TIMER2 (tumor immune estimation resource, version 2; http://timer.cistrome.org/) is a comprehensive resource for systematic analysis of differential gene expression between tumor and adjacent normal tissues [37]. In this study, we input *PXN* in the “Gene_DE” module to evaluate the expression of *PXN* in tumor tissue from different cancer types and in adjacent normal tissues of the TCGA project. The “Immune-Gene” module of TIMER2 was used to evaluate the correlation of *PXN* expression with immune infiltration across all tumors in TCGA project. The “Gene_Corr” module of TIMER2 was employed to investigate the association between *PXN* expression and immune infiltration in different cancer types and in adjacent normal tissues of the TCGA project. Correlations between *PXN* expression and immune infiltration were analyzed statistically using the purity-adjusted partial Spearman’s correlation test. The data was displayed by heatmaps and scatter plots.

### GEPIA2

GEPIA2 (gene expression profiling interactive analysis, version 2; http://gepia2.cancer-pku.cn/#analysis) is an interactive web server for analyzing RNA expression data from tumors and normal samples from TCGA and genotype-tissue expression (GTEx) projects [38]. In this study, the “Expression Analysis-Box Plots” module of GEPIA2 was used to obtain box plots of *PXN* expression between tumor and normal tissues from the GTEx database. We set the *P*-value cutoff = 0.01, log_2_FC (fold change) cutoff = 1, and “Match TCGA normal and GTEx data”. The “Survival Map” module of GEPIA2 was used to obtain the overall survival (OS) and disease-free survival (DFS) significance map data related to *PXN* across all tumors in TCGA. We set cutoff-high (50%) and cutoff-low (50%) values to split the high-expression and low-expression cohorts. The survival data were visualized with hazard ratio, 95% confidence intervals, and log-rank P values. The “Pathological Stage Plot” module of GEPIA2 was used to obtain *PXN* expression in different pathological stages of different tumors in TCGA. The “Similar Gene Detection” module of GEPIA2 was used to identify the top 100 *PXN*-correlated targeting genes from TCGA.

### UALCAN

UALCAN (http://ualcan.path.uab.edu/analysis.html) provides protein expression analysis options using data from TCGA and the CPTAC datasets [39]. In this study, the CPTAC module of UALCAN was used to obtain the expression levels of PXN total protein or phosphoprotein in tumor tissue from different cancers and in adjacent normal tissues. The *P* value cutoff was 0.05.

### Kaplan-Meier plotter

The Kaplan-Meier plotter (http://kmplot.com/analysis/) is a user-friendly website that can assess the effect of genes on different cancer prognoses. We used the Kaplan-Meier plotter to obtain the relationship between the *PXN* gene and a series of analyses of OS, distant metastasis–free survival (DMFS), relapse-free survival (RFS), post-progression survival (PPS), first progression, disease-specific survival (DSS), and progression-free survival (PFS) in different cancers from TCGA (RNA-seq) and GEO (microarray) datasets. The available datasets of five tumors—namely breast, ovarian, lung, gastric, and liver—were split into two groups by setting “autoselect best cutoff”. A log *p*-value < 0.05 was considered statistically significant.

### cBioPortal

cBioPortal (http://www.cbioportal.org) is a comprehensive website that can explore, visualize, and analyze multidimensional cancer genomics data [40]. We obtained the alteration frequency, mutation type, and copy number alteration of the *PXN* gene across all tumors in TCGA via the “Cancer Types Summary” module of cBioPortal. We set the “TCGA Pan Cancer Atlas Studies” in the “Quick select” section to query the genetic alteration characteristics of the *PXN* gene. In addition, the “Comparison” module of cBioPortal was used to obtain data concerning the relationship between *PXN* genetic alterations and prognoses (OS, DSS, PFS, and DFS). The survival results were displayed with log-rank *P* values.

### PhosphoNET

The PhosphoNET website (http://www.phosphonet.ca/) provides information about human phosphorylation sites, their evolutionary conservation, the identities of protein kinases that may target these sites, and related phosphor sites. In this study, we obtained the predicted phosphorylation features of the PXN proteins between primary tumor and normal tissues.

### STRING

The STRING website (https://string-db.org/) is a database of known and predicted protein-protein interactions [41]. Using the STRING website, we obtained a top-50 list of PXN-binding proteins by setting the minimum required interaction score to “Low confidence”, the meaning of network edges to “evidence”, and the max number of interactors to show to “no more than 50 interactors”. The accuracy of the list was consistent with experimental evidence.

### DAVID

DAVID (database for annotation, visualization, and integrated discovery, version 6.8; https://david.ncifcrf.gov/home.jsp) provides a comprehensive, functional annotation tool for investigators to clarify the biological functions of submitted genes [42]. In this study, we conducted an intersection analysis to assess *PXN* binding and interacting genes via a Venn diagram viewer. Using the DAVID tool, we obtained the data as a functional annotation chart. DAVID 6.8 was used for Gene Ontology (GO) enrichment analysis and Kyoto Encyclopedia of Genes and Genomes (KEGG) pathway enrichment analysis of the *PXN* and neighboring genes. The enriched pathways were visualized with the “ggplot2” and “clusterProfiler” R packages.

The data for biological processes, cellular components, and molecular function in the GO enrichment analysis were visualized with the “cnetplots” R package. R-language software (R-4.0.4, 64-bit; https://www.r-project.org/) was used in this analysis. *P* < 0.05 was considered statistically significant.

### Immunohistochemical analysis

HPA (https://www.proteinatlas.org) is a program that aims to map all human proteins in cells, tissues and organs by integrating various omics technologies. HPA was used to illustrate PXN mRNA and protein expression data from different types of human cancers. In addition, we also obtained immunohistochemistry images of PXN proteins from cancer tissues.

### Data availability

The data used to support the findings of this study are included within the article.

## Supplementary Materials

Supplementary Figures
