# Supplementary material for: Pan-cancer analysis reveals an immunological role and prognostic potential of PXN in human cancer
Source: Aging (Albany NY). 2021 Jun 16;13(12):16248–66. doi: 10.18632/aging.203154 (PMC8266322; doi:10.18632/aging.203154)
Supplement: Supplementary Figures [file aging-13-203154-s001.pdf]

SUPPLEMENTARY FIGURES

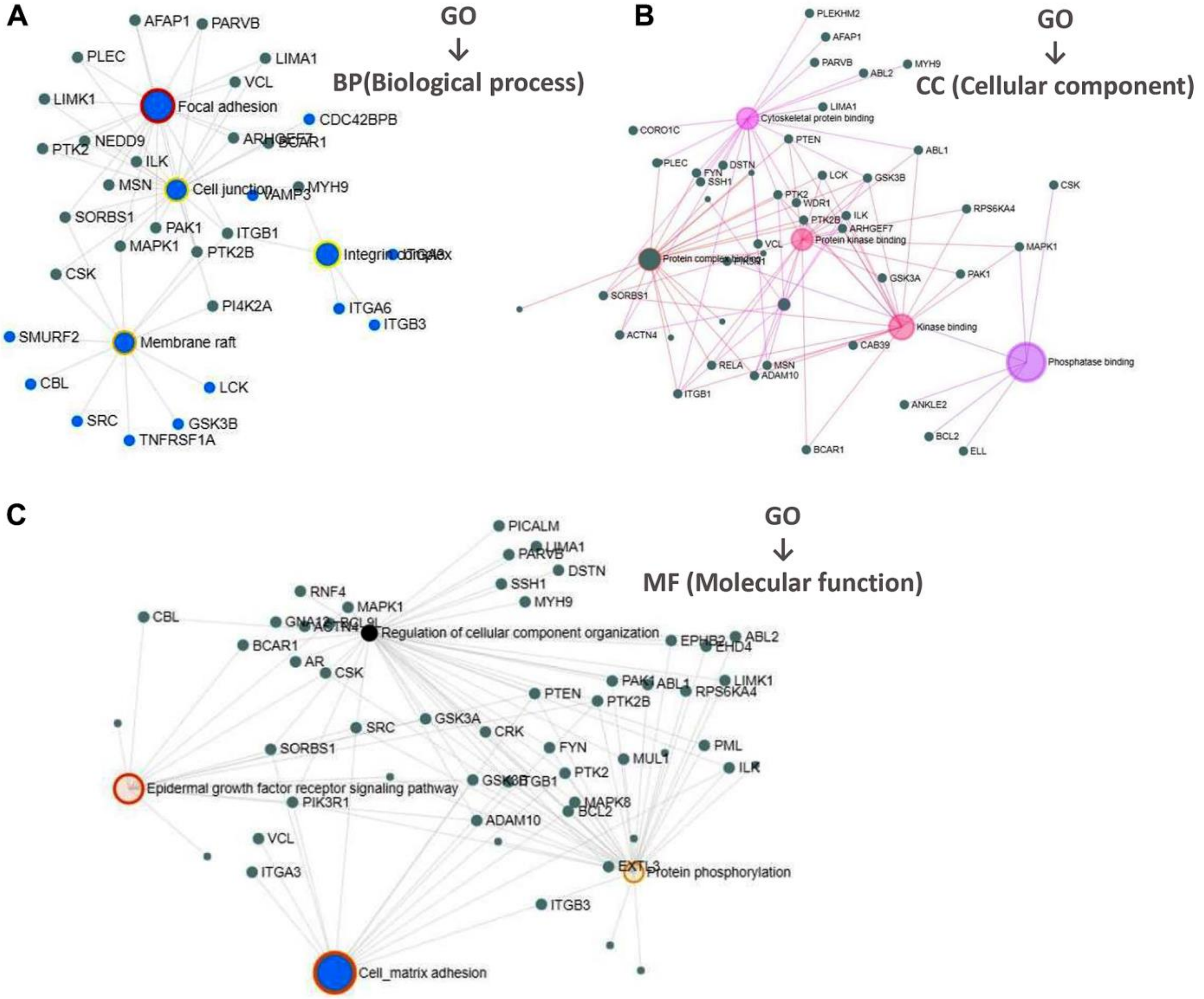

Supplementary Figure 1. Gene Ontology (GO) biological process/cellular component analysis of *PXN*-related genes in tumors. The "cnetplots" models from GO enrichment analysis for (**A**) the biological process, (**B**) the cellular component, and (**C**) the molecular function data are shown.

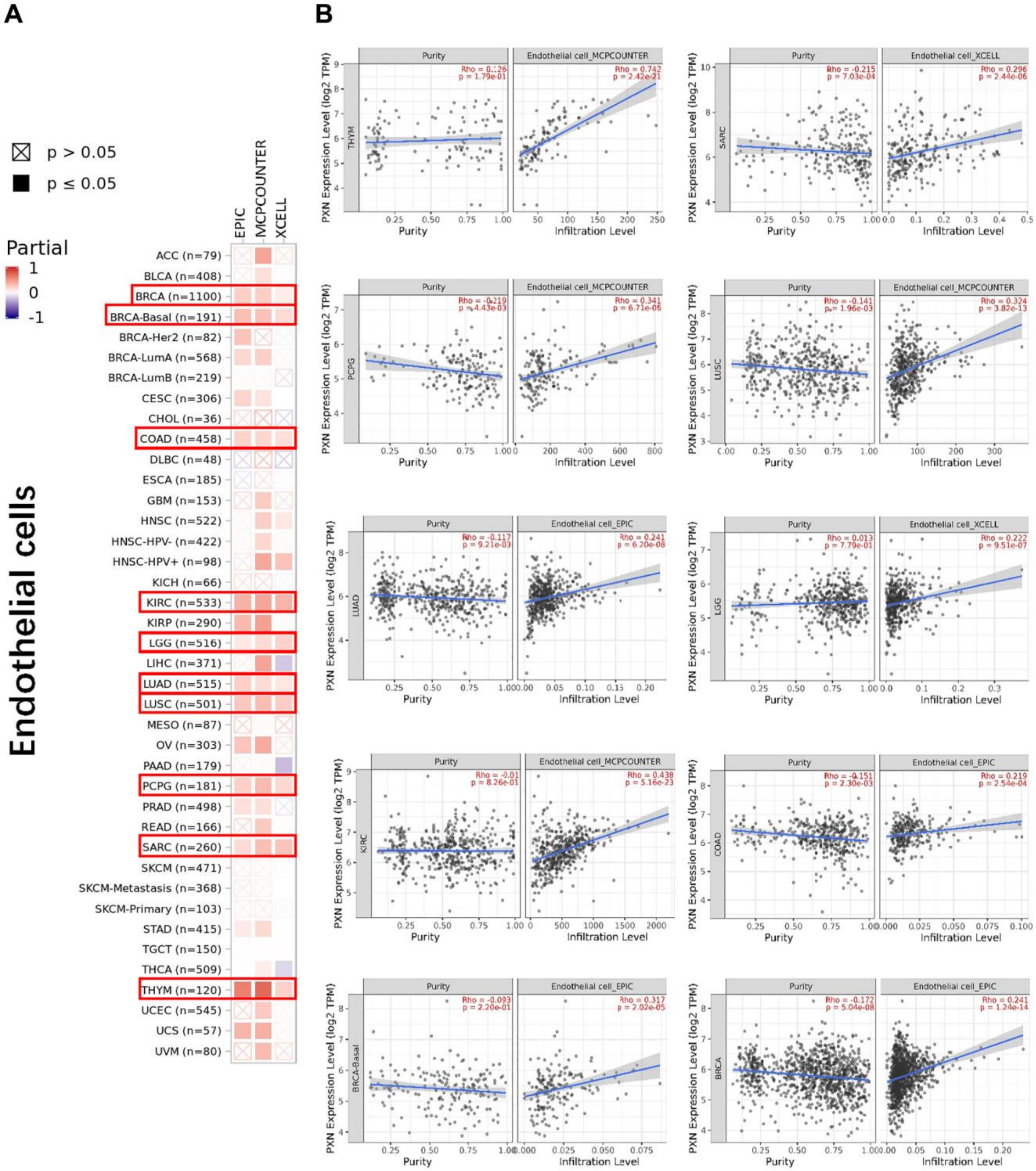

Supplementary Figure 2. Correlation analysis between *PXN* gene expression and immune infiltration of endothelial cells. Different algorithms explored potential correlations of (**A**) the expression level of the *PXN* gene and (**B**) the infiltration level of endothelial cells across all types of cancer in The Cancer Genome Atlas.
